# Diagnosing Allergic Bronchopulmonary Aspergillosis: A Review

**DOI:** 10.7759/cureus.4550

**Published:** 2019-04-27

**Authors:** Avani R Patel, Amar R Patel, Shivank Singh, Shantanu Singh, Imran Khawaja

**Affiliations:** 1 Internal Medicine, Northern California Kaiser Permanente, Fremont, USA; 2 Internal Medicine, Southern Medical University, Guangzhou, CHN; 3 Pulmonary Medicine, Marshall University School of Medicine, Huntington, USA

**Keywords:** allergic bronchopulmonary aspergillosis, aspergillus fumigatus, central bronchiectasis, asthma, cystic fibrosis, eosinophilia, aspergillosis

## Abstract

Dr. Hinson and his colleagues first described allergic bronchopulmonary aspergillosis (ABPA) in 1952. Later in 1977, Rosenberg proposed a diagnostic criteria for ABPA that even today remains widely acknowledged. Despite these steps taken, there still isn't a standardized diagnostic criteria set for ABPA although many have been proposed by various physicians over the years. ABPA is a condition caused by hypersensitivity to Aspergillus fumigatus antigens. It is seen most commonly in patients with either asthma or cystic fibrosis. In susceptible hosts, repeated inhalation of Aspergillus spores can cause an allergic response. Although a standardized diagnostic criteria is re­quired, there is no single test that establishes the diagnosis oth­er than a demonstration of central bronchiectasis (CB) with nor­mal tapering bronchi, a feature that is still considered pathognomonic of ABPA. Because of lack of standardized diagnostic criteria and screening, even today ABPA is under diagnosed and often times treatment for it is delayed. This can lead to complications in patients like pulmonary fibrosis, bronchiectasis with chronic sputum production, and increasingly severe persistent asthma with loss of lung function. For this alone, it becomes imperative that the diagnostic criteria guidelines need to be reviewed and standardized preferably with the help of larger research studies. In the following review article, we address the epidemiology, pathophysiology, and the current cumulative view regarding the diagnosis of ABPA.

## Introduction and background

Aspergillosis is a condition in which Aspergillus fungi infect tissues. Aspergillosis affecting the respiratory tract can have several manifesta­tions that range from hypersensitivity disorders to rapidly inva­sive disseminated disease, all of which can be classified within three distinct categories [1-2]. They are allergic aspergillosis, saprophytic colonization, and invasive aspergillosis [3]. Allergic aspergillosis can be further subdivided into three different forms. They include allergic bron­chopulmonary aspergillosis (ABPA), Aspergillus-induced asthma (AIA), and allergic Aspergillus si­nusitis (AAS) [3]. ABPA is a condition caused by a hypersensitivity reaction to Aspergillus antigens [3]. ABPA is classified differently depending on the criteria that the patient meets [4]. Asthma patients who meet the minimum criteria but do not have central or peripheral bronchiectasis are classified as serologic ABPA or ABPA-seropositive (ABPA-S) [5-7]. Patients who meet the minimum criteria for ABPA and also have central bronchiectasis are classified as ABPA-central bronchiectasis (ABPA-CB) [5-7]. Lastly, patients who have severe asthma and sensitivity to fungi but do not meet the criteria for ABPA are classified as severe asthma associated with fungal sensitivity (SAFS) [5-7]. In susceptible hosts, an allergic response is evoked by repeated inhalation of Aspergillus spores [3]. These spores get trapped in the thick sputum of asthmatic subjects causing a cascade of inflammatory reactions that can result in ABPA [3]. ABPA is found most commonly in atopic patients, or in patients with cystic fibrosis or asthma. The following review article addresses ABPA and its epidemiology, pathophysiology, and diagnostic criteria.

## Review

Epidemiology

ABPA affects approximately 1% through 15% of cystic fibrosis patients [8]. One study calculated that 2.5% of adults who have asthma also have ABPA, which is approximately 4.8 million people worldwide [9]. From 1983 through 1986, Greenberger and Patterson found an ABPA prevalence of 6% from their 531 asthmatic patients [10]. In other studies, ABPA was detected in 25% to 37% of asthmatics with a positive skin prick test to Aspergillus fumigatus (Af) [11].

Pathophysiology

**Figure 1:**
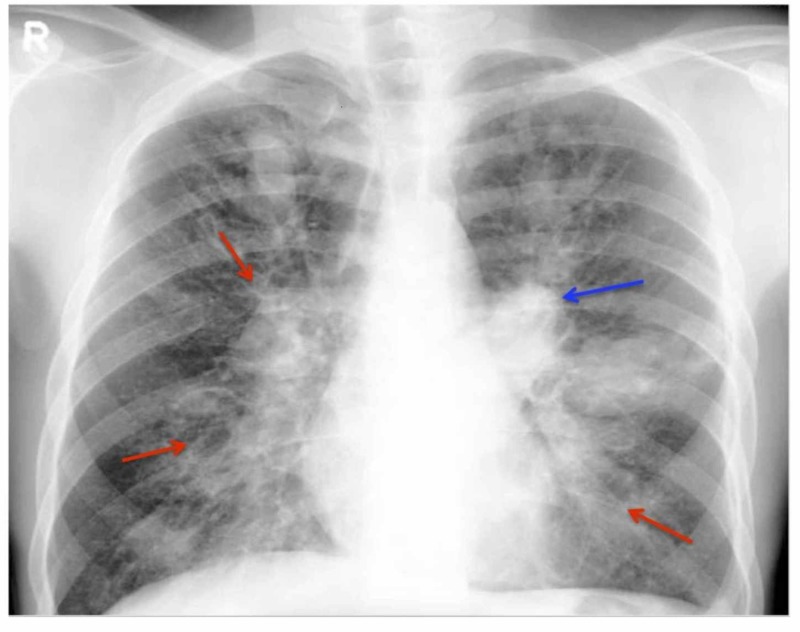
Plain Chest Radiograph The chest radiograph of an allergic bronchopulmonary aspergillosis (ABPA) patient shown with left-sided perihilar opacity (blue arrow) along with non-homogeneous infiltrates (transient pulmonary infiltrates indicated by red arrows) in all zones of both lung fields, seen in acute and remission stage of ABPA [3].

**Figure 2:**
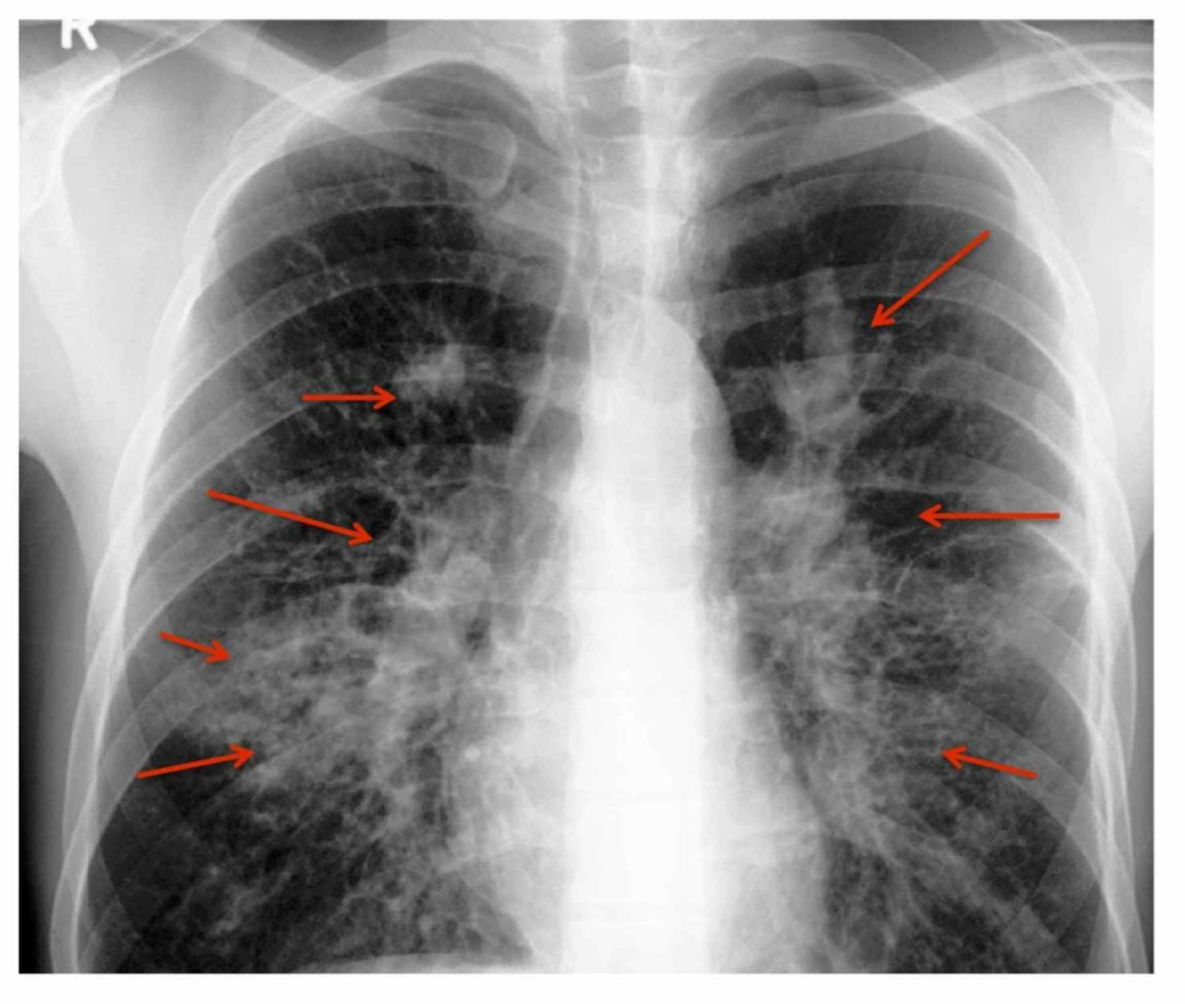
Chest Radiograph of the Same Patient Four Months Later Chest radiograph showed spontaneous resolution of left-sided perihilar opacity with an increase in non-homogenous infiltrates (red arrows) [3].

ABPA is caused by hypersensitivity to Aspergillus antigens [3]. In susceptible hosts, repeated inhalation of Aspergillus spores can cause an allergic response. This reaction is mainly an immunoglobulin E (IgE) mediated hypersensitivity reaction [3]. Both type III and type IV, immunoglobulin G (IgG) mediated immune complex and cell-mediated hypersensitivity reactions have also been seen [12]. ABPA is divided into five stages (i) acute, (ii) remission, (iii) exacerbation, (iv) corticosteroid-dependent asthma, and (v) fibrotic lung disease (Table 1) [12-13]. The chest radiograph of the patient on admission (diagnosed with ABPA) and four months later is shown in Figures 1-2.

**Table 1:** Conventional Staging in ABPA ABPA: allergic bronchopulmonary aspergillosis; IgE: immunoglobulin E; CT: computed tomography.

Conventional Staging
Stage I Acute: The patient is diagnosed with ABPA. All typical features such as aspergillus-specific IgE, radiological abnormalities (Figures 1-2), peripheral blood eosinophilia, and aspergillus-specific serum precipitins [3].
Stage II Remission: Asymptomatic patient with underlying controlled asthma with no new radiological infiltrates and no rise in total IgE for a minimum of six months [3].
Stage III Exacerbation: New pulmonary infiltrates appear on chest radiograph with peripheral blood eosinophilia and double the remission level IgE levels [3].
Stage IV Corticosteroid Dependent Asthma: Patients become dependent on corticosteroid treatment and are unable to completely taper off from them [3].
Stage V Fibrotic Lung Disease: Chest radiograph and CT scans will show irreversible fibrosis and chronic cavitation. Despite this, serological parameters are usually negative [3].

Diagnostic criteria

**Figure 3:**
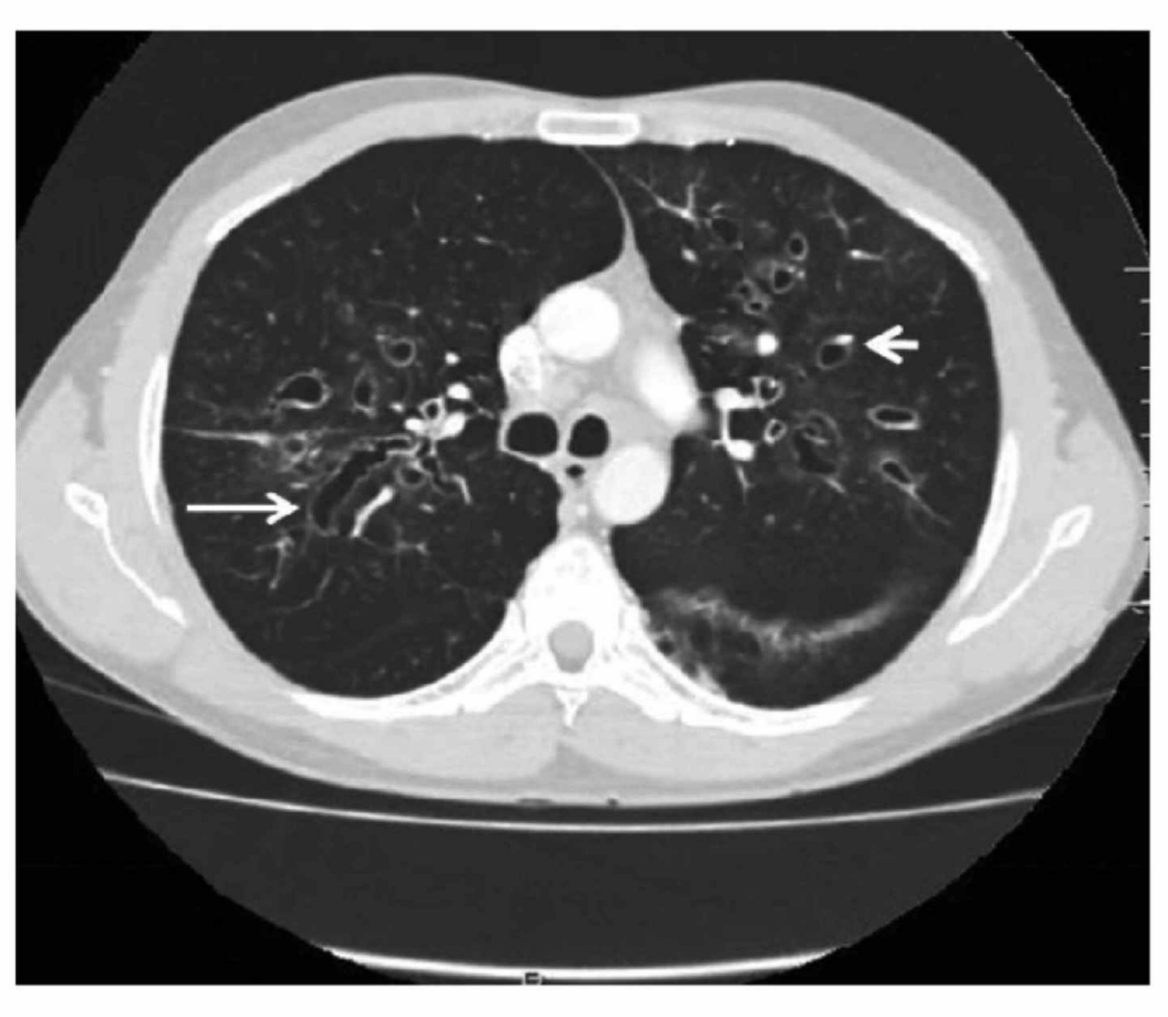
Computed Tomography (CT) of the Thorax CT showing ‘signet ring’ (short, thick arrow) and ‘string of pearls’ (long, thin arrow) appearances, indicative of central bronchiectasis. Mucoid impaction and dilated bronchi are also seen [3].

**Figure 4:**
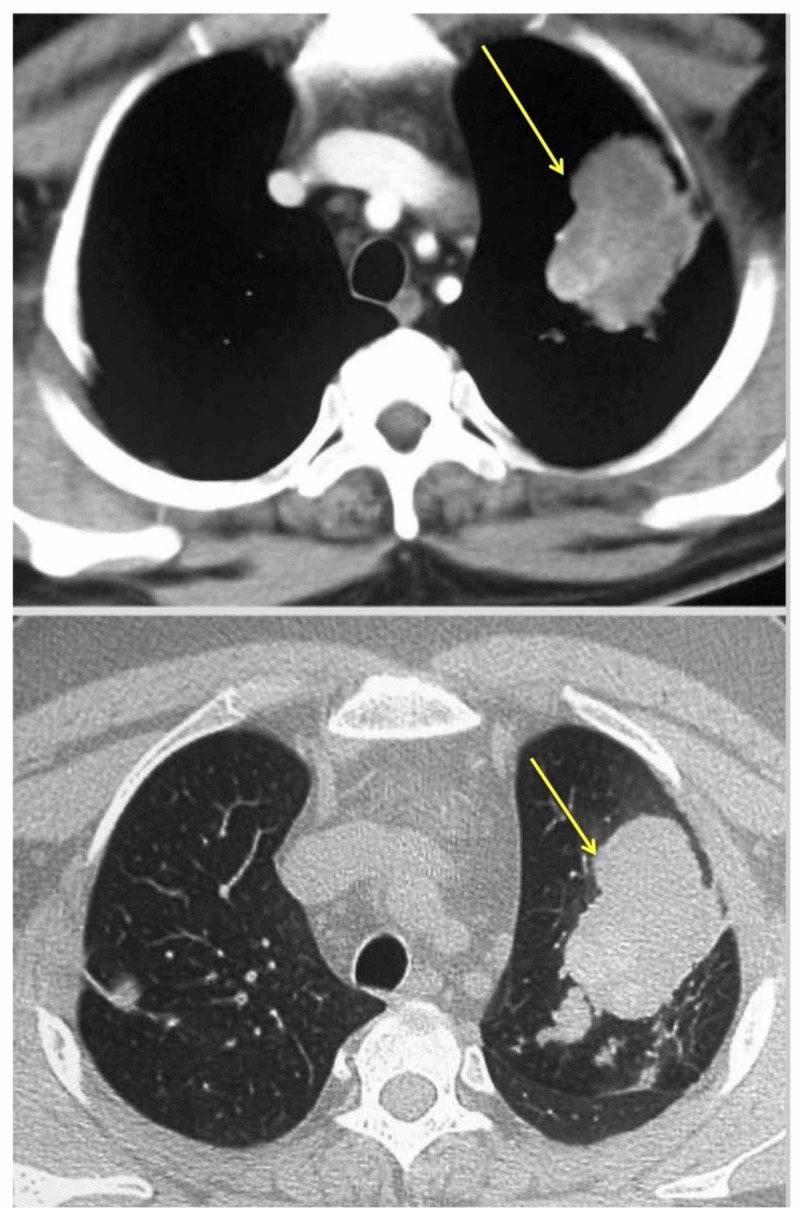
High-resolution Computed Tomography (HRCT) of the Thorax HRCT of the thorax (mediastinal window and corresponding section on the lung window) showing high attenuation mucous (HAM) impaction (yellow arrow) [3]. This is considered pathognomic for allergic bron­chopulmonary aspergillosis (ABPA) by the International Society for Human and Animal Mycology (ISHAM) working group [3].

ABPA was first discovered by Hinson in 1952 when he reported and reviewed eight ABPA cases [14]. Years later in 1968, ABPA was first diagnosed in the United States and only afterwards became recognized on a global scale [13,15]. Over the years, the key diagnostic features have been standardized [15-16]. Because of the lack of consensus, there have been many proposed criteria over the years (Table 2). Although a set of criteria is re­quired, there is no single test that establishes the diagnosis oth­er than a demonstration of central bronchiectasis (CB) with nor­mal tapering bronchi, a feature still considered pathognomonic of ABPA (Figure 3) [15-17]. On high-resolution computed tomography (CT), high-attenuation mucous (HAM) plugs were also reported in 28% of patients with ABPA (Figure 4) [18]. The International Society for Human and Animal Mycology (ISHAM) working group has highlighted this finding and con­siders HAM a pathognomonic feature of ABPA [18].

**Table 2:** Diagnostic Criteria for ABPA ABPA: allergic bronchopulmonary aspergillosis; Af: Aspergillus fumigatus; CB: central bronchiectasis; CF: cystic fibrosis; IgE: immunoglobulin E; IgG: immunoglobulin G; ISHAM: International Society for Human and Animal Mycology.

Name	Diagnostic Criteria for ABPA
1977, Rosenberg-Patterson Criteria [15, 16]	Major Criteria: (1) asthma, (2) presence of fleeting or fixed pulmonary opacities on chest radiograph, (3) immediate cutaneous hypersensitivity reaction to Af, (4) total serum IgE elevated, more than 1000 IU/mL, (5) precipitating antibodies against Af, (6) peripheral blood eosinophilia, (7) central or proximal bronchiectasis with normal tapering of distal bronchi
	Minor Criteria: (1) golden brown sputum plugs in expectorant, (2) positive sputum culture for aspergillus species, (3) late (arthus-type) skin reactivity to Af
1999, ABPA in CF [19]	Presence of two out of following three: (1) immediate cutaneous hypersensitivity reaction to Af, (2) presence of precipitating antibodies to Af, (3) elevated total IgE levels more than 1000 IU/mL
	Plus at least two of following six: (1) bronchoconstriction, (2) eosinophil count more than 1000/μL, (3) history of pulmonary opacities on chest radiograph, (4) elevated IgE or IgG antibodies to Af, (5) Af in sputum smear or sputum culture, (6) response to steroids
2002, Minimum Essential Criteria [20]	Criteria: (1) asthma, (2) immediate cutaneous hypersensitivity reaction to Af, (3) total serum IgE elevated more than 1000 ng/mL (417 kU/L), (4) elevated IgE and IgG antibodies to Af, (5) CB in absence of distal bronchiectasis
2012, Minimum Criteria and Additional Criteria [4]	Minimum Criteria: (1) patients with asthma or cystic fibrosis, (2) worsening lung function, (3) positive skin prick test with aspergillus species, (4) total serum IgE greater than 1000 ng/mL (416 IU/mL), (5) increased aspergillus species-specific IgE and IgG antibodies, (6) infiltrates noted on chest radiography
	Additional Criteria: (1) increase in serum eosinophilia when the patient is not on corticosteroids ( more than 400 eosinophils/μL), (2) aspergillus species-specific precipitating antibodies, (3) central bronchiectasis, (4) aspergillus species-specific containing mucus plugs
2013, Truly Minimal Criteria [21]	Criteria: (1) asthma, (2) immediate cutaneous hypersensitivity reaction to Af, (3) total serum IgE elevated more than 1000 ng/mL (417 kU/L), (4) CB in absence of distal bronchiectasis
2013, ISHAM Working Group [22]	Predisposing Conditions: (1) asthma, (2) CF
	Obligatory Criteria (both need to be present): (1) type I aspergillus skin test positive (immediate cutaneous hypersensitivity reaction to Af) or elevated IgE levels against Af, (2) elevated total IgE levels more than 1000 IU/mL (unless all other criteria is met, then total IgE levels can be less than 1000 IU/mL)
	Other Criteria (two out of three at least): (1) presence of IgG antibodies against Af or precipitating antibodies, (2) presence of fleeting or fixed pulmonary opacities on chest radiograph consist with ABPA, (3) eosinophil count more than 500 cells/μL in steroid naïve patient (may be a historical value)

Rosenberg-Patterson Criteria

In 1977, Rosenberg and Patterson proposed a diagnostic criteria divided into major and minor criteria. All eight major criteria may not be found at all times [3]. Some of the features may be present only during the acute (stage 1) or the exacerbation (stage 3) stages [3]. The major criteria consisted of patients diagnosed with asthma, the presence of pulmonary opacities on chest radiographs, immediate cutaneous reactivity to Af, the serum IgE being more than 1000 IU/mL, precipitating antibodies against Af, peripheral blood eosinophilia, and central or peripheral bronchiectasis with normal tapering of distal bronchi [15-16]. The minor criteria consisted of finding golden brown sputum plugs in expectorant, a positive sputum culture for Aspergillus species, and a late (arthus-type) skin reactivity to Af [15-16].

ABPA in Cystic Fibrosis

In 1999, Geller proposed a two-part diagnostic criteria. The first part needed two confirmed points out of three [19]. These were an immediate cutaneous reactivity to Af, the presence of precipitating antibodies to Af, and the elevated total IgE levels being more than 1000 IU/mL [19]. The second part needed two confirmed points out of the following six. These were bronchoconstriction, an eosinophil count more than 1000/μL, a history of pulmonary opacities on chest radiograph, elevated IgE or IgG antibodies to Af, the presence of Af in sputum smears or cultures, and finally an observed response to steroids [19].

Minimum Essential Criteria

In 2002, Greenberger proposed a criteria that consisted of five points. These are a confirmed diagnosis of asthma, an immediate cutaneous reactivity to Af, a total serum IgE more than 1000 ng/mL (417 kU/L), elevated IgE and IgG to Af, and central bronchiectasis in absence of distal bronchiectasis [20].

Minimum Criteria and Additional Criteria 

In 2012, Knutsen updated the ABPA criteria with a minimum criteria and an additional criteria [4]. The minimum criteria consists of patients with asthma or cystic fibrosis, worsening lung function, a positive skin prick test with Aspergillus species, a total serum IgE greater than 1000 ng/mL (416 IU/mL), increased Aspergillus species-specific IgE and IgG antibodies, and finally, infiltrates noted on the chest radiograph [4]. The additional criteria included an increase in serum eosinophilia when the patient is not on corticosteroids (more than 400 eosinophils/μL), Aspergillus species-specific precipitating antibodies, central bronchiectasis, and lastly, Aspergillus species-specific containing mucus plugs [4].

Truly Minimal Criteria

In 2013, Greenberger established a second set of diagnostic criteria. It contained four points [21]. They are a previous diagnosis of asthma, an immediate cutaneous reactivity to Af, an elevated serum IgE more than 1000 ng/mL (417 kU/L), and central bronchiectasis in absence of distal bronchiectasis [21].

ISHAM Working Group

In 2013, the ISHAM working group developed their own criteria for ABPA. It was divided into predisposing conditions like asthma or cystic fibrosis. The next section was the obligatory criteria which consisted of two points, both of which need to be present [22]. They are an immediate cutaneous reactivity to Af or elevated IgE levels directed against Af, and elevated total IgE levels more than 1000 IU/mL [22]. The other criteria consisted of three points, in which two of three must be present. They are the presence of IgG antibodies against Af, the presence of pulmonary opacities on chest radiograph, and lastly an eosinophil more than 500 cells/μL in steroid naïve patient [22].

## Conclusions

The material reviewed in this paper focuses on the diagnosis of ABPA. It goes into detail regarding the epidemiology, the pathophysiology, and the diagnostic criteria seen with ABPA. Despite these key points being addressed, more and larger studies are needed to create a standardized criteria that can be used worldwide to diagnose ABPA. Although there have been many proposed criteria over the years, it is imperative that a standardized one is created that can be utilized worldwide. This is a review article for busy, practicing physicians to have a cumulative view of our current situation regarding where the medical community is in our understanding of the diagnosis of ABPA.
